# The social and scientific values that shape national climate scenarios: a comparison of the Netherlands, Switzerland and the UK

**DOI:** 10.1007/s10113-017-1155-z

**Published:** 2017-04-26

**Authors:** Maurice Skelton, James J. Porter, Suraje Dessai, David N. Bresch, Reto Knutti

**Affiliations:** 1 Department of Environmental Systems Science, Institute for Environmental Decisions, ETH Zürich, Zürich, Switzerland; 2 Department of Environmental Systems Science, Institute for Atmospheric and Climate Science, ETH Zürich, Zürich, Switzerland; 3 Sustainability Research Institute and ESRC Centre for Climate Change Economics and Policy, School of Earth and Environment, University of Leeds, Leeds, LS2 9JT UK; 4 Institute of Work Psychology, Sheffield University Management School, Sheffield, UK

**Keywords:** Climate scenarios, Adaptation, Decision-making, Co-production, Civic epistemology

## Abstract

This paper seeks to understand why climate information is produced differently from country to country. To do this, we critically examined and compared the social and scientific values that shaped the production of three national climate scenarios in the Netherlands, Switzerland and the UK. A comparative analysis of documentary materials and expert interviews linked to the climate scenarios was performed. Our findings reveal a new typology of use-inspired research in climate science for decision-making: (i) innovators, where the advancement of science is the main objective; (ii) consolidators, where knowledge exchanges and networks are prioritised; and (iii) collaborators, where the needs of users are put first and foremost. These different values over what constitutes ‘good’ science for decision-making are mirrored in the way users were involved in the production process: (i) elicitation, where scientists have privileged decision-making power; (ii) representation, where multiple organisations mediate on behalf of individual users; and (iii) participation, where a multitude of users interact with scientists in an equal partnership. These differences help explain why climate knowledge gains its credibility and legitimacy differently even when the information itself might not be judged as salient and usable. If the push to deliberately co-produce climate knowledge is not sensitive to the national civic epistemology at play in each country, scientist–user interactions may fail to deliver more ‘usable’ climate information.

## Introduction

Extreme weather events cause damage, disruption and loss of life across the world. As the present climate changes, extreme events like floods and heatwaves are likely to become more frequent and intense (IPCC 2014). To adapt, society needs to better understand how the climate might change in the future, together with the associated risks. By showing how the temperature may change or rainfall patterns shift over the next century, climate information can help inform adaptation planning and decision-making. National climate scenarios have taken up this challenge. They paint a picture of how the future climate may change for a country, on the basis of a set of greenhouse emission pathways. As Hulme and Dessai (2008) explain, scenarios have a long and varied history, originating in military strategy and planning in the 1950s and expanded by the energy industry in the 1970s before becoming a common tool for decision-making in government.^1^ National climate scenarios, as a result, have become influential decision support tools for adaptation in the UK (Jenkins et al. 2009), Switzerland (CH2011 2011), Germany (DWD 2012), South Africa (DEA 2013), Ireland (Gleeson et al. 2013), the Netherlands (KNMI 2014a), the USA (Melillo et al. 2014) and Australia (CSIRO and Bureau of Meteorology 2015), amongst others.

Yet, climate information often remains unused because it is seen to be too complex, not sufficiently relevant or unusable. To narrow this ‘usability gap’ (Lemos et al. 2012), scholars have focused their attention on how to bring scientists and users together to deliberately co-produce climate information (Meadow et al. 2015; Dilling and Lemos 2011). If scientists understand what climate information is needed and, in turn, users understand what scientists can provide, delivering relevant and usable science could face less barriers; it is argued (Lemos and Rood 2010). How this should be done is unclear, however. Tangney and Howes (2016) have shown that the credibility, legitimacy and saliency of climate information are viewed differently from one country to the next. Different political cultures and scientific values affect how climate information is produced and the extent to which users are involved (Hanger et al. 2013; Beck 2012; Jasanoff 2005; Shackley 2001). This is because, in part, the way science is publicly acknowledged, circulated and legitimised in each country reflects its own ‘civic epistemology’ (Jasanoff 2005). That is the process by which countries ‘assess the rationality and robustness of claims that seek to order their lives’ (ibid). While greater scientist–user interactions should be encouraged, those advocating co-production need to be aware of the existing social and political cultures they are intervening in. If not handled carefully, efforts to co-produce climate knowledge may amplify the voice of some at the expense of others with different needs (Klenk and Meehan 2015).

The relationship between the state and science differs from country to country. In the UK, scientific expertise and political authority are separated to deliver *objective* and *rational* knowledge to support *pragmatic empiricist* policy-making (Mahony and Hulme 2016; Tangney 2016; Rothstein et al. 2013; Jasanoff 2005). Yet, this same expertise is often funded by UK government departments with their own agendas (Tangney 2016; Steynor et al. 2012). Other countries have very different set-ups. Neither Switzerland nor the Netherlands has a majority government. Decisions have to be consensual. Otherwise, nothing proceeds. Inclusion of the political, scientific, public and private minorities is common. It has been argued that compromises can be found easier through a closed nature of inclusion and a lack of transparency in how decisions are made, as actors are able to negotiate (and concede) without public scrutiny (Hermann et al. 2016; Andeweg and Irwin 2005). Differences between Dutch and Swiss political cultures do exist, though. In the Netherlands, the policy-making process is more participatory in that it includes political elites, interest groups and individual citizens (Andeweg and Irwin 2005; van der Brugge et al. 2005). In Switzerland, by contrast, different representatives from politics, public administrations and interest groups mediate policies between themselves, with the Swiss electorate called on to decide issues in referendums if a consensus cannot be reached (Hermann et al. 2016).

In this paper, we seek to understand why climate scenarios are produced differently from country to country by examining the social and scientific values that shape it. To do this, we focus on the experiences of suppliers of climate information, namely scientists and advisors, responsible for delivering climate scenarios whose voices are critical yet too often silent in co-productionist studies (Cvitanovic et al. 2015). We performed a comparative analysis of three countries—the Netherlands, Switzerland and the UK—which share a number of similarities in modelling capacities yet chose to design their climate scenarios in very different ways. After explaining our methods and data, we compare the modelling approaches, institutional arrangements and climate information provided in each country. We then investigate the different motivations for producing climate scenarios, before we turn to the different scientist–user interactions. To close, we develop a typology to explain the differences in how and why the climate scenarios took the particular shape they did.

## Data and methods

To understand how climate scenarios are produced and, importantly, why they differ from one country to another, we adopted a case study approach to examine the recent efforts of climate scientists in the Netherlands, Switzerland and the UK. We chose these case studies because they share a number of similarities and differences. Each country has a history of developing climate scenarios, enjoys well-funded climate programmes and makes use of state-of-the-art computing facilities and expertise, yet each differs in the modelling approaches taken and the degree to which users were involved.

To examine these case studies in greater depth, we brought together the findings from two methods. First, we conducted a desk-based search to identify documents (e.g. briefing reports, technical summaries, guidance notes) relating to the release of each set of climate scenarios. These documents provide a public record as to why modelling decisions were taken, how users participated in the process and the reasoning behind different presentational styles in each country. A total of 37 documents were imported to MAXQDA—a qualitative coding software—and analysed (*n* = 12, KNMI’14; *n* = 13, CH2011; *n* = 12, UKCP09). We then manually coded the documents to identify emergent themes on a range of topics from the treatment of uncertainty, involvement of users and lessons learnt.

Second, we conducted semi-structured interviews (*n* = 10) with climate scientists and advisors responsible for delivering the Dutch and Swiss climate scenarios during 2015/2016. We supplemented this data with five interviews performed with actors involved in the UK’s climate scenarios in mid-2013 (Porter and Dessai 2017). Whenever possible, interviews were held face-to-face in participants’ offices or via Skype. We adopted a conversational approach, which allowed people to express their views and experiences on aspects of the production process not covered in the official documentation we analysed. To that end, we asked: Why are climate scenarios needed? Who was involved in the production process, and what role did they play? And, to what extent were users involved, and what did they contribute? All the interviews were digitally recorded (with consent) and transcribed using an intelligent verbatim transcription approach—omitting filler words or hesitations (Hadley 2015). Once the transcripts were imported into MAXQDA, we manually coded the responses to identify emergent themes including modelling decisions, user engagement and institutional relationships.

To introduce greater rigour to our findings, we triangulated the codes from both datasets to understand where the greatest agreement, or disagreements, existed.

## Context: how do the British, Dutch and Swiss climate scenarios compare?

Despite only a few years separating the release of the British, Dutch and Swiss climate scenarios, they differ in a number of ways (see Table 1). Briefly introducing each of the climate scenarios below, we highlight how these differences are not only concerned with the way climate change was assessed, or the actors involved, but also how each country presents climate information.

**Table 1:** A broad comparison of British, Dutch and Swiss climate scenarios, 2009–2014

	UK—UKCP09 land scenarios	Switzerland—CH2011	Netherlands—KNMI’14
Previous national climate scenarios	CCIRG91; CCIRG96; UKCIP98; UKCIP02	CH2007	Buishand and Tank 1996; WB21; KNMI’06; 2009 supplements to KNMI’06
Scientific bodies	Met Office Hadley Centre (MOHC)	Federal Office of Meteorology and Climatology MeteoSwiss; Swiss Federal Institute of Technology Zurich (ETH), Center for Climate Systems Modeling (C2SM)	Royal Netherlands Meteorological Institute (KNMI)
Boundary organisations	UK Climate Impacts Programme (UKCIP)	ProClim Forum for Climate and Global Change	None
Funders	Department for Environment, Food and Rural Affairs (Defra); Department for Energy and Climate Change (DECC)	ETH and MeteoSwiss through in-kind contributions; smaller financial contributions by the Swiss Federal Office of Energy (SFOE); Federal Office for the Environment (FOEN); through C2SM by Empa, Agroscope Reckenholz-Tänikon, ETH Zurich Foundation	Ministry of Infrastructure and the Environment
Advisory bodies	Steering Group (strategic: MOHC and Defra); Project Management Group (operational: MOHC); User Panel (consultative: UKCIP)	Coordination Group (strategic and advisory)	International Advisory Board (8 scientific members from other European climate research institutions)
Review Process	Method reviewed by International Review Group with 6 members from the UK, the USA and Canada (UKCP09 (Review Group 2009)), reports reviewed by user panel members	Report reviewed by climate scientists (11 named + anonymous). Methods and models had already been published or were in press with academic journals (Buser et al. 2009; Fischer et al. 2011)	Internal review from the Advisory Board with the methods published in an academic journal (Lenderink et al. 2014), summary report reviewed by selected users
Emission scenarios used	3 emission scenarios (A1F1, A1B and B1)	3 emission scenarios (A1B, A2 and RCP3PD)	4 (2 driving variables: global temp and air circulation; 2 conditions: high or low)
Ensemble	Perturbed physics ensemble (PPE); multi-model ensemble (MME)	MME	Initial state perturbation for 8 EC-Earth integrations; MME
Data source	280 global climate model (GCM) runs with HadSM3; 13 GCM HadCM3 runs; 11 regional climate model (RCM) HadRM3 variants	8 GCMs; RCMs from ENSEMBLES: *n* = 20 up to 2050; *n* = 14 up to 2100; *n* = 10 for the daily data for meteorological stations up to 2100	Downscaling of 8 EC-Earth GCM runs with RACMO2 RCM; 250 GCM calculations of Coupled Model Intercomparison Project Phase 5 (CMIP5)
Regional differentiation	25-km^2^ grid cells (434 selectable land grid squares); 23 river-basin regions; 16 administrative regions	Averaged over 3 regions (without the Alpine region)	None; apart from a qualitative differentiation for temperature
Time horizons	2020s, 2050s and 2080s available as monthly, seasonal and annual 30-year means/ probabilities (daily and hourly via the weather generator)	2020–2049, 2045–2074 and 2070–2099 available as seasonal ranges (daily via raw data)	2030s (combining all 4 scenarios), 2050s and 2080s available at seasonal and annual ranges, daily via raw data
Climate variables	25 (e.g. temperature, precipitation, sea-level rise; cloudiness; solar radiation)	10 (*n* = 2 quantitative: temperature and precipitation; *n* = 8 qualitative: summer heat waves, intense rainfall, droughts, etc.)	12 (e.g. temperature, precipitation, sea-level rise, fog) stated as 22 indicators (e.g. mean, daily maximum, number of days ≥20 mm)
Electronic resources	User interface website with a visualiser; product reports (e.g. marine, land, observations, weather generator; *n* = 518 pages)	Website to download climate scenario reports (e.g. summary and scientific, *n* = 94 pages), raw data and subsequently provided extensions (no user interface)	Website to download scenario reports (e.g. brochure and scientific; *n* = 156 pages) and raw data with all indicators at station scale (no user interface)

### UK’s climate scenarios: the UKCP09 land scenarios

After 7 years of work, the UK Met Office Hadley Centre released the world’s first set of probabilistic climate scenarios: the UKCP09 land scenarios,^2^ in 2009. This modelling endeavour was largely driven by the Met Office Hadley Centre, whilst the UK Climate Impacts Programme (UKCIP) managed the user engagement. Funded by the UK Government, the climate scenarios serve as an ‘input to the difficult choices that planners and other decision-makers will need to make, in sectors such as transport, healthcare, water resources, and coastal defences’ by giving users the freedom to choose the scale, time period and thresholds corresponding to their risk tolerance and appetite (Jenkins et al. 2009).

**Fig. 1:**
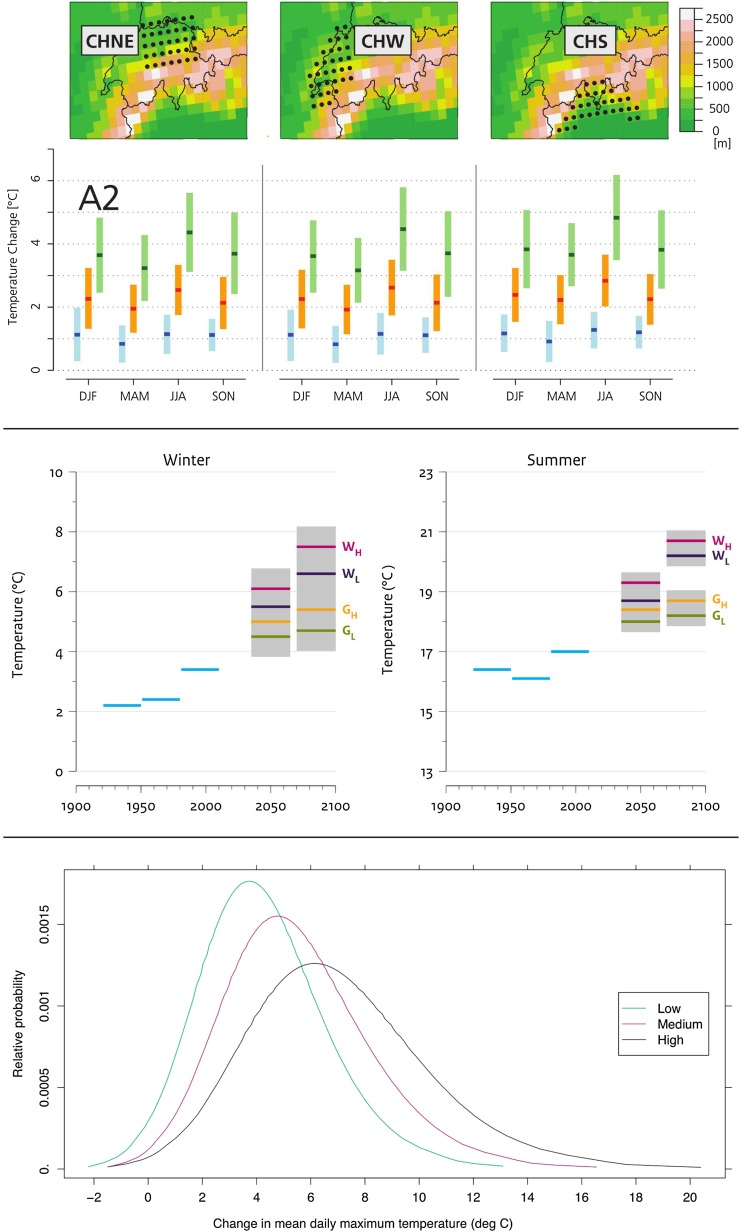
Comparison of the visuals of the British, Swiss and Dutch climate scenarios, 2009–2014. *Top* CH2011 divides Switzerland into three climatic areas with corresponding seasonal ranges for three future time periods. The example shows temperature changes under emission scenario A2. *Middle* KNMI’14 only visualises winter and summer temperature and precipitation changes to increase legibility, combining all four scenarios with three historical averages. Data for autumn, spring and the natural variability are available only through a table. *Bottom* UKCP09 visualises likely changes as probability density functions (PDFs) for each of the three emission scenarios. This graph holds no temporal information—for each of the climate variables, time periods, grid points and regions, such a graph is available online. The example indicates changes in summer-mean daily maximum temperature in South East England for the 2080s (sources: CH2011 2011; KNMI 2014a; Jenkins et al. 2009)

A major focus for UKCP09’s climate scenarios was its effort to account for the inevitable uncertainty around future climate change. Probability distribution functions are provided to indicate the plausible range of climate change under a particular emission scenario—with an expression of how strongly different outcomes are supported by different lines of evidence (e.g. climate science, observations and expert judgement) (see Fig. 1; Jenkins et al. 2009). For instance, users can assess the likelihood that temperatures will increase by more than 3 °C in London in the 2080s relative to the 1961–1990 base period. A large number of climate simulations were run to capture structural model uncertainties, accounting for different climate models’ ability to replicate key aspects of current and future climate change. To do this, a perturbed physics ensemble with the Met Office Hadley Centre’s own climate model was combined with a multi-model ensemble from other modelling centres through a novel and complex (yet as a consequence somewhat contentious) Bayesian approach that used a statistical climate model emulator (see Frigg et al. 2015; Parker 2010).

The climate scenarios are given at a resolution of 25 km^2^ over land or as averages for administrative regions and river basins. Confidence varies within the data, however. It is highest at the continental scale and lowest at the local scale, which interests users most (Porter and Dessai 2016). Users can choose from seven time periods, with overlapping 30-year windows spanning 2010 to 2099. Users, in turn, are also encouraged to work with all three emission scenarios: high, medium and low, to learn the full extent of possible changes (Jenkins et al. 2009). The climate scenarios are available free of charge via three formats: (1) key findings (headline messages, maps and graphs), (2) published materials (reports, guidance and case studies for various sectors) and (3) customisable outputs (raw data via the user interface website) (Steynor et al. 2012). After the launch, there were updates to the climate scenarios; for instance, *spatially coherent projections* were provided so that users could combine results from grid boxes to create spatial information.

### Switzerland’s climate scenarios: CH2011

Released in 2011, the Swiss climate scenario CH2011 marked the completion of a joint science-led initiative by the Institute for Atmospheric and Climate Science at the Swiss Federal Institute of Technology Zurich (ETH) and the Federal Office for Meteorology and Climatology MeteoSwiss, with contributions from the Center for Climate Systems Modeling (C2SM, a research collaboration housed at ETH), the National Centre of Competence in Research Climate (NCCR Climate, a major Swiss research grant housed at the University of Berne) and the Swiss Advisory Body on Climate Change (OcCC, housed at the civil society organisation ProClim). CH2011 provides a new assessment detailing how Switzerland’s climate may change over the next century. Delivered without a mandate but officially sanctioned once completed,^3^ the climate scenarios provide a ‘coherent’ basis to develop ‘climate change impact studies… addressing ecologic, economic, and social’ consequences to inform ‘climate adaptation and mitigation strategies’ (CH2011 2011).

CH2011 climate scenarios are ‘based on a new generation of global and European-scale regional climate models’ (CH2011 2011). Switzerland does not have its own global climate model, but ETH contributed to the regional climate modelling COSMO-CLM community project. This means CH2011’s ‘model data have been provided by several international projects’ instead (CH2011 2011). Climate simulations from the ENSEMBLES project (van der Linden and Mitchell 2009), as well as studies and assessments from the Intergovernmental Panel on Climate Change (IPCC), were used. New, but importantly peer-reviewed, statistical methods were used to generate multi-model ensemble estimates of changes and associated uncertainties. Probability statements as in the IPCC (i.e. *likely* indicating at least two in three chances of the value falling in the given range), but no PDFs, are assigned to temperature and precipitation only, under three emission scenarios (two non-intervention and one climate stabilisation) to give users an indication of the likely direction of change (e.g. summer rainfall likely to decrease by 6–23% for 2060 in the western part of Switzerland in the A2 scenario) (CH2011 2011).

The climate scenarios were aggregated spatially into three broad regions with much of the Alps excluded, as its topographical complexity raised concerns over how to reliably interpret the model results (CH2011 2011). Projected changes over the twenty-first century are broken into three time periods (2020–2049, 2045–2074 and 2070–2099) and are available as seasonal and daily ranges. The CH2011 climate scenarios can be accessed freely for research, education and commercial purposes, by visiting the website and downloading the individual datasets (e.g. regional scenarios at daily resolution) or by requesting the published reports for the main findings. Following the release of CH2011, two extensions were published, providing annual averages, climate scenarios for the Alpine region and station-scale daily data for all three emission scenarios.

### Netherlands’ climate scenarios: KNMI’14

The Royal Netherlands Meteorological Institute (KNMI) issued the country’s most recent climate scenarios in 2014: KNMI’14. Funded by the government, the climate scenarios ‘will be used [by decision makers] to map the impacts of climate change… [and] evaluate the importance and the urgency of climate adaptation measures’ for building coastal defences, healthcare, city planning and nature conservation (KNMI 2014b).

A defining feature of KNMI’14 is the use of four scenarios to visualise how future climate may change around 2050 and 2085 (see Fig. 1). Each scenario differs in terms of the amount of global warming (moderate or warm) and possible changes in air circulation (low or high). Around 2085 (2071–2100), under the G_L_ scenario (low air circulation change, low global temperature rise), annual mean temperature is projected to be 1.3 °C warmer than the reference period (1981–2010) whereas, under the W_H_ scenario (high air circulation change, high global temperature rise), it could be 3.7 °C warmer. To obtain a range (e.g. for summer daily maximum extremes), KNMI’14 provides the currently observed natural variability onto which users can superimpose the future climate change signal to derive future upper and lower bounds. These scenarios show a single spatial scale: the whole of the Netherlands. This is because ‘any attempt to make climate predictions at a relatively small spatial scale such as the Netherlands or even Western Europe for multiple decades ahead cannot be expected to lead to skilful results’ (KNMI 2014b).

Eight initial-state perturbed climate simulations with the community global climate model EC-Earth (co-supported by the Dutch) and their own regional climate model RACMO2 were performed. These were then supplemented with a multi-model ensemble from the Coupled Model Intercomparison Project Phase 5 (CMIP5) (WCRP 2010). Users are able to access the KNMI’14 climate scenarios free of charge by downloading the published reports or requesting the dataset directly from KNMI. After KNMI’14 was published, an inconsistency in the W_L_ scenario for 2085 was found which prompted KNMI to issue a rectified version in late 2015.

### Key differences between the British, Swiss and Dutch climate scenarios

We found four key differences in how the British, Dutch and Swiss scientists approached the production and dissemination of their climate scenarios. Simply put, these differences include (i) modelling capacities, (ii) treatment and communication of uncertainty, (iii) the actors involved and (iv) access to the data.

First, whereas the British and Dutch have their own climate models, the Swiss rely on utilising modelling efforts of others. In turn, the British climate scenarios took a more computationally demanding and complex modelling approach than its counterparts. Second, this gave rise to the British incorporating the structural model error explicitly, with the help of a Bayesian statistical model. This inclusion of model uncertainties broadens the spread of model simulations, which they communicated as PDFs for each emission scenario. In theory, the PDFs incorporate the expert judgment needed to interpret the information *correctly*. The Swiss and Dutch followed the IPCC approach whereby interpretation and use of model results need expert judgement. But, they did so differently. The Swiss used Bayesian statistics to estimate PDFs but communicated only a lower, medium and upper value as representative plausible outcomes for each emission scenario. Because of the Netherlands’ high vulnerability to coastal flooding and the profound implications on most national activities, changes in wind direction have been judged as an additional key uncertainty. Coastal defences, among other adaptation options, need to incorporate both increased storm surges due to wind as well as sea-level rise due to emission scenarios. To incorporate this, the Dutch assessed and communicated their uncertainties along these two dimensions, providing single figures for each of the four storylines.

Third, the Dutch kept the entire modelling and user engagement within a single organisation: KNMI, whilst the British and Swiss included various, institutionally distinct and physically distant, actors for these tasks. For instance, the CH2011 community comprised multiple institutions, with some scientists asked to represent the views of multiple actors (and users) simultaneously. Lastly, although the British provide users with all the output data and guidance on potential limitations, the Dutch and Swiss restricted what information users received. The Swiss withheld parts of the data relating to the Alps due its topographical complexity and the Dutch aggregated the data into two driving variables, air circulation change and temperature. These different epistemological preferences affect the reasoning behind how climate scenarios are done in the first place.

## What is the purpose of climate scenarios?

Two main reasons were cited by all three sets of scientists as to why they felt it was important to produce and disseminate climate scenarios. First, in order to take well-informed adaptation and mitigation decisions, a single coherent body of locally relevant scientific information is needed. Second, such exercises can help advance scientific understanding through the development of new methods, computing power and working relationships. Although the three case studies share these two objectives, our research suggests that they were prioritised, understood and acted upon differently.

### Informing climate adaptation and mitigation decision-making

All interviewed climate scientists agreed that their country needed its own set of climate scenarios because decision-makers are primarily ‘interested in their local patch’ (UKCP09 scientist 5) and because weather patterns are different from one place to another (KNMI’14 scientist 1). The IPCC assessment reports and its regional climate scenario chapter (Christensen et al. 2007) are simply ‘too coarse’ to inform local or sector-based adaptation decision-making (CH2011 scientist 2).

A growing user base, with evolving requirements, has also led to ‘many requests for additional information and guidance’ such as the inclusion of more climate variables, extreme weather events and regional details that larger-scale climate scenarios cannot provide (KNMI 2014b). Servicing the informational needs of these users is a major purpose of climate scenarios. All the scientists shared this conviction and went to great lengths to stress how they wanted their work not only to be ‘useful’ to decision-makers but also importantly ‘used’ by them (CH2011 scientist 4).

National policies added further support for use-inspired science. All three countries have enacted legislation requiring climate scenarios to inform national-scale policy-making as well as local-scale decision-making in public and private organisations. Only in Switzerland have climate scenarios emerged without a governmental mandate (only to be officially approved prior to publication) (CH2011 scientist 2). Yet, in each case, efforts to co-produce climate scenarios have been skewed in favour of scientists who retained power over ‘what these scenarios look like’ or ‘when to provide these scenarios’ (KNMI’14 advisor 1).

Another key purpose of climate scenarios for KNMI scientists was to initiate a ‘paradigm shift’ in how users think (KNMI 2014b). Moving away from responses based on experiences of ‘past climatic events’, users should instead anticipate ‘possible future conditions’ for decisions today (KNMI 2014b). UKCP09 scientists also felt that climate scenarios helped reaffirm the different roles and responsibilities of those involved in adaptation decision-making:

It’s not the climate scientist’s responsibility to provide a golden number [for users] and accept that risk [for it]. Because [scientists] can only provide what is the best science at the time, and make all the uncertainties available before saying ‘Okay, this is our best estimate, so take from that what you can’. And then it’s over to users as to how they use it (UKCP09 advisor 1).

Some users may, however, struggle with this epistemological position. Users may become frustrated or confused if they identify and manage their risks differently to how the climate scenarios have prescribed them, especially if they prefer to work with single figures rather than a range (or PDFs). As Porter and Dessai (2017) argue, UKCP09 scientists often see users as miniature versions of themselves—mini-mes—who struggle to understand why anyone would not want to use probabilistic information, which, for them, represents the *best* science available. This can lead to tensions when users who ‘rely on a definitive answer being provided for them’ fail to receive one (UKCP09 advisor 1). By contrast, KNMI’14 scientists felt one of the main purposes of climate scenarios was to engage as many people, from different backgrounds with different interests, as possible so as to actively avoid giving users multiple, perhaps conflicting, outputs (KNMI’14 scientist 2). For each variable, users were given only a single figure (average) for its four scenarios. That is, for a variable of interest, users must compare four averages (one for each of the four scenarios) in order to see if there are differences or trends, and their size, between the four scenarios. This was less likely to be misinterpreted or cause confusion; it is argued (KNMI 2014b).

### Advancing scientific knowledge

One, if not the main, driver for developing each set of climate scenarios was the opportunity to advance scientific knowledge. However, the three groups of scientists interpreted their intellectual contribution differently. For instance, KMNI’14 and CH2011 aimed to improve and consolidate the range of scientific information used in decision-making for their respective countries (CH2011 scientist 4), whereas the UKCP09 climate scenarios wanted to develop a ‘new method for quantifying uncertainty’ with international reach too (UKCP09 scientist 2).

Newly developed methods, improved computing power and recently released model runs (e.g. CMIP5), alongside the availability of new observation datasets, were all cited as reasons for producing climate scenarios. For KNMI’14 scientists, advances in climate modelling opened up a new dialogue with users including water managers and health specialists over ‘what could or couldn’t be done’, so that users helped prioritise the scientific work (KNMI’14 scientist 2). It also allowed KNMI’14 scientists to test if the predecessor, KNMI’06, underestimated the impact of air circulation patterns on temperature rise (KNMI 2014b). Interestingly, KNMI’14 scientists were ‘a little disappointed with the final result [due to] the similarity of the outcomes’ between KNMI’06 and KNMI’14 (KNMI’14 scientist 1). Whilst KNMI’14 scientists reiterated their primary goal to improve the usability and use of the climate scenarios, the satisfaction derived from being the first to discover some scientific novelty is still important. Researchers’ desire to advance scientific knowledge about climate and explore new ways of thinking about climate decisions (probabilities), it seems, can conflict with the more pragmatic needs of users (i.e. highly robust information presented in familiar ways) to enable effective adaptation planning. Therefore, the extent to which co-production will help to resolve these tensions or exacerbate them further as those involved in the supplying and demanding climate information became more frustrated with each other is unclear.

For CH2011 scientists, the need to advance scientific understanding via a new set of climate scenarios was expressed differently. Already serving as IPCC lead authors but lacking the modelling resources enjoyed by other countries (Brönnimann et al. 2014), the CH2011 climate scenarios strengthened old and encouraged new collaborations between Swiss research institutions (CH2011 advisor 1). It brought researchers and (scientific) users ‘to one table’ where everyone could discuss how the modelling should be done (CH2011 scientist 4). ‘There wasn’t always a consensus within the group’ because the complex topography of the Swiss Alps presents challenges for modelling. But, by ‘bringing together the different institutions’, the Swiss climate science community was able to speak with ‘one voice’ for the first time and created the momentum to fund future climate scenarios, as well as political support to establish the Swiss National Centre for Climate Services (CH2011 scientist 4).

UKCP09 scientists differ from their KNMI’14 and CH2011 counterparts in how they understand and, in turn, acted upon the need to both advance scientific knowledge and inform adaptation decision-making. For KNMI’14 and CH2011 scientists, the two objectives can sometimes be incompatible whereas UKCP09 scientists felt that they went hand-in-hand. UKCP09 scientists assumed that if users want to make ‘reliable, robust, and relevant’ decisions, ‘they need the best science’ available (UKCP09 scientist 3). Better science, it seems, equals better decisions (see Porter and Dessai 2017). What constitutes *good* science for decision-making for the British and Dutch scientists is understood differently, however. In contrast to the single figures provided in KNMI’14, UKCP09 quantifies climate variables’ ranges so that users can decide about the level of risk they want to manage. Where multi-model ensembles have conventionally been used to assess uncertainty, UKCP09 scientists felt this method failed to capture the full range of uncertainties (Porter and Dessai 2016). By developing their own method, not only would they make a significant intellectual contribution to quantifying model uncertainties but they could also meet the institutional–political goals set by the Met Office, the Department for Environment, Food and Rural Affairs (Defra) and the now disbanded Department of Energy and Climate Change (DECC) to produce world-leading science, with the potential to influence the IPCC process (UKCP09 scientist 2).

### Different understandings, different priorities

All three sets of scientists were fully committed to informing adaptation decisions and advancing scientific understandings yet interpreted these commitments differently. For CH2011 scientists, priority was given to assembling a consistent evidence base that spoke with one voice. To do this, the effort was focused on improving working relationships and intellectual exchanges to advance scientific capacities. For KNMI’14 scientists, a major driver was the need to change how people think and act in relation to climate change. Advances in climate modelling certainly aided this process but were not the sole catalyst. For UKCP09 scientists, efforts to quantify uncertainty were underpinned by the assumption that users need the *best science* possible. Practical or application-based considerations inevitably took a backseat to intellectual contributions and the pursuit of curiosity-driven science. These different understandings of the purpose of climate scenarios affect the way users are involved in the process and the extent to which they are listened to.

## How involved did scientists think users were in producing the climate scenarios?

Our research suggests that all three sets of climate scenarios differed considerably in the extent to which they involved users, what they expected them to contribute and even whom they thought the user was in the first place. Together, these differences have had a marked effect on the particular form taken by the British, Dutch and Swiss climate scenarios. For instance, how model uncertainty was quantified (cf. UKCP09 vs. KMNI’14) is based on a series of assumptions about the capacity of users to work through and make sense of complex information. However, narrowly defined perceptions of users and their needs have seriously diluted the stated commitment to co-produce national climate scenarios.

### Scientists’ perceptions of users

Without exception, the official documents issued for all three sets of climate scenarios paint a very broad picture of potential users. From actors interested in digging down and exploring the data to those interested only in the headline messages, the scientists hoped that their climate scenarios will be used by the widest audience possible. In other words, the climate scenarios should not become the exclusive preserve of a small group of actors. This manifests itself differently in each country. Where the KNMI’14 and CH2011 climate scenarios aimed to inform decisions in sectors from water, healthcare, agriculture and transport to infrastructure, UKCP09 went even further by subdividing the users within these sectors into three categories: researchers, decision-makers and communicators (Steynor et al. 2012). Simply put, all three climate scenarios should officially cater to different users, all with different needs.

Few of the scientists interviewed shared that view, however. CH2011 scientists, for instance, felt the end users would be either impact modellers or government officials (CH2011 scientist 1). Previous experiences from the last climate scenarios, CH2007, and the government agenda to develop a national adaptation strategy, informed this view. Yet, misunderstandings over what users need and what scientists think is useful (see Lemos et al. 2012) soon developed. CH2011 scientists realised they had ‘produced far more information than [government officials] could use’ or make sense of (CH2011 scientist 1). Lacking the time and resources to work through the probability statements provided, government officials were forced to simplify the climate information they used. A ‘user bubble’ of likeminded individuals—impact modellers—consulted by the CH2011 scientists meant they had, unintentionally, overestimated the capacity of non-quantitative users (Liniger 2015). Upon reflection, CH2011 scientists told us that while it was fairly intuitive to identify which sectors might be interested in using climate scenarios, it remained a mystery how the climate scenarios would actually be used or what users needed from them (CH2011 scientist 3).

UKCP09 scientists, similarly, were confident that they ‘knew what users needed’ (UKCP09 scientist 1). With over 25 years of experience developing climate scenarios (e.g. LINK project, CCIRG, UKCIP), scientists had formed close working relationships with several users: impact modellers, water managers and consultants (Porter and Dessai 2017, 2016; Hulme and Dessai 2008). All of these users share certain characteristics. They are highly numerate, motivated and knowledgeable actors. These characteristics were woven into the fabric of the new climate scenarios. That is, UKCP09 requires users to have already assessed their vulnerability to climate change themselves to be able to use PDFs (Jenkins et al. 2009). A persistent criticism, though, is that potential users without the time, resources or capacity to make sense of their vulnerabilities can find themselves excluded (Frigg et al. 2015; Tang and Dessai 2012). Indeed, UKCP09 scientists were warned against defining the user too narrowly (Steynor et al. 2012). Very late in the process, the government funder, Defra, pushed for the climate scenarios to be opened up to ‘as many people as possible’ to avoid satisfying only a single type of user (UKCP09 scientist 2).

KNMI’14 scientists did things differently. They already knew water managers were the primary user of the previous climate scenarios, KNMI’06 (KNMI’14 scientist 1). Unlike their CH2011 or UKCP09 counterparts, ‘the first meeting of the [KNMI’14] project team was on user requirements’ (KNMI’14 advisor 1). Put differently, KNMI’14 scientists believe that limiting the volume of (undigested) information given to users, and the choices they have to make, improves the accessibility and understanding of the climate scenarios. Asking users to focus on four storylines places less demands on their time and requires only a basic level of understanding, initially at least. KNMI’14 scientists, therefore, imagined different users with different needs and capacities (KNMI’14 scientist 2).

### Scientists’ perceptions of user interactions

Despite initial reluctance from some scientists to involve the intended and favoured users, by the end, a closer working relationship between the two became highly valued. Scientists concerned over lack of time or the right skills to engage with favoured users soon realised that with a better understanding of how climate information is used, and therein what users need, they could make a ‘few small changes with immediate impact’ (UKCP09 scientist 1). The only way to do this was for scientists and users to meet face-to-face, something the UK has been doing since the early 1990s (see Hulme and Dessai 2008). Yet, all three sets of climate scientists held very different views on the interaction format and the extent to which users were listened to.

CH2011 scientists told us that users ‘weren’t involved as much as they would have liked’ (CH2011 scientist 1). Both a lack of ‘funding’ and official ‘mandate’ was cited as major barriers (CH2011 scientist 2). Efforts were made to ensure the voice of users was heard, nonetheless, although ‘we didn’t do a full user survey… [canvassing only impact modellers] we still had a good impression [of]… what users needed’ (CH2011 scientist 4). Moreover, when a coordination group was set up to oversee the production of the climate scenarios, two of the six seats were filled by user representatives. Mirroring the political culture of Swiss collegiality, the coordination group required members to reach decisions collectively. Yet, it was not always easy for user representatives to relay the ‘heterogeneous needs’ of users (CH2011 advisor 1). As a consequence, this institutionalised the user bubble rather than challenged it (Liniger 2015). Users were only introduced en masse just ‘before the report was released’ where ‘talks and events’ were held so that everyone ‘who should know about [the climate scenarios] did know about them in advance’ (CH2011 scientist 4). However, not only is awareness different from engagement, but the introduction of users at such a late stage restricts what they can, and are willing to, contribute and articulate.

KNMI’14 and UKCP09 scientists both conducted surveys with users from previous versions of their climate scenarios and ran workshops to understand how user needs have changed. A long ‘shopping list’ of requirements was identified but was interpreted and acted upon differently. For instance, the ‘explicit presentation of [model] uncertainties and assumptions behind [them], easier access [to the data], and higher temporal and spatial resolution [data]’ was flagged by both projects (Steynor et al. 2012; see also Bessembinder et al. 2011). Whereas this confirmed UKCP09 scientists’ need to advance science linearly (UKCP09 scientist 1), KNMI’14 scientists felt a closer dialogue was needed to dispel the ‘you ask, we deliver’ paradigm in the hope that users reconsider their requests (KNMI’14 scientist 3). Indeed, KNMI’14 scientists raised concerns about the methods to elicit user needs. For them, surveys risk closing down fruitful conversations about user needs, and therein, fail to understand how, or why, users actually use climate information:

You cannot just go to users once and ask them for feedback. You need to have regular contact, continuous contact, over a long time to get really useful feedback. It’s not just asking ‘what do you want?’ and then giving it to them… many users want to do something with climate adaptation but don’t know exactly what that is or how to do it… so it’s important to know how they use climate data (KNMI’14 advisor 2).

To encourage as much interaction as possible, many face-to-face meetings between scientists and users were organised (KNMI’14 advisor 2). Two communication experts were hired to get users more involved instead of ‘just listening to talks’ (KNMI’14 scientist 2). ‘Light workshops with standing tables’ mixing scientists and users with ‘only six people around each table… to make it easy to ask questions’ were used (KNMI’14 advisor 2). This set-up helped scientists to better understand how climate information is used and, in turn, what users need. It also opened up conversations over ‘the advantages and disadvantages of probability distributions and the way uncertainties are presented’ and differences between what is doable and what is desirable by getting users to think more reflexively about ‘their list of requests’ (Bessembinder et al. 2011). ‘That discussion and dialogue between users and KNMI staff really was the main contribution of the three years of work. Much more so than the analysis of the data and the climate scenarios’ (KNMI’14 scientist 2).

UKCP09 scientists, by contrast, were less enthusiastic about interacting with users than their KNMI’14 counterparts. That reluctance was due, in part, to different ideas about the roles and responsibilities of scientists (Porter and Dessai 2017). As Mahony and Hulme (2016) observe, UKCP09 scientists saw their job as pushing the boundaries of climate modelling and solving practical problems to inform governmental policy and decision-making, while organisations like UKCIP should engage users because they possess the ‘right skills and time’ to do so (UKCP09 scientist 2). Part of the British political culture of evidence-based decision-making serves to reinforce this separation of scientists and users, in order to preserve the integrity and authority of expert knowledge, on the one hand, and a top–down hierarchy between the two is maintained, on the other (Tangney and Howes 2016). That said, 3 years after the modelling began, the UKCP09 project was reorganised, and UKCIP’s idea of bringing users and scientists together via a user panel was achieved with the support of the funder, Defra (UKCIP 2006). Practical concerns were raised, such as the number of users involved, how regularly (or when) to consult them and how to weigh their contributions equally. For instance, there is the risk that ‘users who [are] able to eloquently express their needs or regularly attended meetings’ gain greater attention or have ‘undue influence’ on the output of the user panel (Steynor et al. 2012). Yet, user input for the climate scenarios was highly constrained. Modelling decisions had gone beyond the point of being reversed (cf. Corner et al. 2012). Users were left to comment on ‘presentation issues’ over the spatial aggregation of the outputs (e.g. 25-km^2^ grid cells vs. river basins) rather than discussing how to model uncertainty differently (UKCP09 advisor 2). The lecture-like set-up with ‘talk after talk’ focused on selling the climate scenarios to users (UKCP09 scientist 2).

### Doing things together

The motivation, intensity and format of the scientist–user interaction were different across the three countries. The ‘you ask, we deliver’ paradigm was used strategically in UKCP09 to support their scientific work but dispelled by KNMI as they felt that a discussion on how climate data is used was more fruitful. In addition, the timing was problematic for both the British and Swiss climate scenarios. Users engaged with UKCP09 only after the major decisions have already been taken (and the funder Defra stepped in), and in CH2011, the interaction was confined to awareness. At best, this limits what contributions users can make, and at worst, it can lead to frustration and disengagement.

This limited interaction was partly accepted because British and Swiss scientists felt they *knew* who the user was. In the Swiss case, this happened through official channels between federal offices or past research collaborations. In the UK, the Met Office had been working with users alongside UKCIP since 1997, so UKCP09 scientists felt that they had already developed considerable (tacit and explicit) knowledge of users. Yet, the users that UKCIP formally introduced to the Met Office often asked highly technical questions that UKCIP could not answer itself. That filtering process (unintentionally) skewed how Met Office scientists saw *users* (Porter and Dessai 2017). This only confirmed what UKCP09 scientists thought users wanted. In both the Swiss and British cases, an early and broader user engagement might have flagged up some warning signs over what scientists thought users needed and what users wanted. For KNMI’14 scientists, the shift in water management practices was only the starting point. It served to question preconceptions of users in other sectors too and avoid falling prey to confirmation bias.

## Discussion

Our comparative analysis reveals that climate scenarios are strongly influenced by the civic epistemology of each country, which defines who has a say, what roles scientists and users should play and how the two interact. Internal disagreements on methodological aspects, communication and target users exist but are often masked by the prevailing science–society relations.

As shown in Table 2, what constitutes good science for decision-making is understood differently from one country to the next: *consolidator* (CH2011), *innovator* (UKCP09) and *collaborator* (KNMI’14). Simply put, the Swiss are more conservative. They emphasise the need for tried-and-tested methods that have been peer-reviewed (e.g. scientific consensus) whereas the British were more adventurous. They applied a new, largely untested, method for quantifying model uncertainties on the assumption that users need this information to adapt effectively (Porter and Dessai 2017). The Dutch have mixed established methods with novel ones when culturally acceptable (Enserink et al. 2013; van der Brugge et al. 2005; see also Dilling and Berggren 2015). A major concern here is when a mismatch develops between what makes science good for decision-making in the eyes of scientists compared to what makes science good for decision-making for the more pragmatic needs of users. For instance, UKCP09 was too complex for some users (Tang and Dessai 2012) and too bold for some scientists (Frigg et al. 2015), which has impeded its uptake and use.

**Table 2:** Comparison of characteristics of climate science and user engagement of between UKCP09, CH2011 and KNMI’14 according to the two proposed typologies: the first ‘typology of scientists’ capturing features important to (climate) science aimed at decision-making, and a corresponding ‘typology of user engagement’ on how users were involved and listened to

	Innovator—UKCP09 land scenarios	Consolidator—CH2011	Collaborator—KNMI’14
Number of institutions	2 plus 1 active funder	5 or more (some producers have several affiliations)	1 (funder not active)
Tasks of institutions	Distinctive—but diverging	Less clear—but with goal consensus	Distinctive
Institutions’ physical distance	High—several hours journey	Medium—all in Zurich, with 1 exception	Low—same building
Scientific innovation	Very important—driving motivation	Less important	Intermediate—but high if it benefits users
Scientific consensus orientation	Less important—UKCP09 needed to be ‘novel’	High—driving motivation, with emphasis on peer-reviewed, consensual findings	Intermediate—but high if it benefits users
	Elicitation—UKCP09 land scenarios	Representation—CH2011	Participation—KNMI’14
Number of users involved	40	2–5 (depends if MeteoSwiss producers are counted as users)	At least 70 users, more likely to be 100+
Scientists’ inclination to engage with users	Initially low, raising to medium	High with the representatives, low with individuals	High—driving motivation, with a particular focus on interaction with individuals
Start and duration of engagement	Formalised user elicitation began after all modelling decisions taken; met every 3 months over a period of 3 years	With representatives: from the start until the end, with lots of discussions; individual users were notified but not engaged	Throughout the whole process
Prior knowledge required for use	High—very numerate users	Medium—user has to be able to read and understand complex topics	Low—entry barrier for use is held as low as possible (no ranges, etc.)

Our ‘typology of use-inspired research’, shown in Table 2, also develops other social science work on the values and assumptions that shape atmospheric science. For Shackley (2001), climate modelling centres judge good scientific practice differently in response to different institutional–political priorities. A modelling hierarchy can emerge where greater modelling complexity is assumed to provide greater realism and better decision-making (Mahony and Hulme 2016; Shackley et al. 1998; Shackley and Wynne 1995). While UKCP09 has gone down the modelling complexity route, CH2011 and KNMI’14 question what value is added by this.

All three climate scenarios differ considerably in how users were engaged, which speaks to different types of user–scientist interaction (Table 2): *participation* (KNMI’14), *elicitation* (UKCP09) and *representation* (CH2011). While the Dutch KNMI involved a large number of users in the production process, the British and Swiss limited interactions to retain power over production. Knowingly or not, science is socially responsive. Different funding mechanisms, institutional arrangements, epistemic cultures and preferences to risk affect what knowledge is produced (by whom and how it is used). This develops Jasanoff’s (2005) civic epistemology work that climate science comes to reflect wider societal concerns expressed through national politics (e.g. Swiss consensus building, Dutch inclusiveness and UK expert authority; see also Beck 2012).

Our two proposed typologies bring a much needed socio-political context into the ‘knowledge systems’ framework by Cash et al. (2003). Where the ‘typology of scientific enterprise’ characterises how judgements of good science give rise to *credible* information, the ‘typology of user interaction’ explains what is involved in producing *legitimate* knowledge for decision-making. Through the culturally situated production of climate information, the scientific output is expected to be *salient* (i.e. relevant) for governmental decision-making—a key argument of the civic epistemologies (Jasanoff 2005). Relevance and usability of scientific information are not synonyms, however. Lemos et al. (2012) argue that usability is high when information is tailored to needs and capacities of users, a quality achieved through co-production where scientists listen to users and respond to their needs. Our results support this proposition: UKCP09 only included sophisticated and numerate members in their user panel while KNMI’14 included a broad user base. The climate scenarios from both countries essentially served only the users involved in their (co-)production.

We conclude, therefore, that several future discussions are needed to better understand the different cultures for producing climate information. First, funders and scholars who advocate for scientists to co-produce climate information with users need to be sensitive to, and reflect upon, the existing social and political cultures that shape climate information. Generalising case studies into *best practices* or one-size-fits-all lists disregard the cultural sensitivities, which influence the successful uptake of climate information (Webber 2015). Second, further research is needed on the role government-approved climate information plays in narrowing the usability gap. Civic epistemologies profoundly influence how *usable* climate information is constructed by both scientists and users. Can political cultures similar to the UK produce knowledge that serves a larger user base with different capacities—but still be salient for government policy-making? What challenges does this present? And, how do users with simpler needs judge the credibility and legitimacy of salient knowledge, in the absence of governmental approval?

Third, the growing number of climate knowledge providers, brokers and specialists has led to calls for increased harmonisation of modelling methods, climate variables and climate service institutions across Europe. Although this promises greater consistency and comparability, as well as lower financial costs, many national governments are ‘keen on exercising and strengthening their own epistemic sovereignty’ rather than offloading power to supra-national climate service institutions (Mahony and Hulme 2016). It is unclear how well European climate knowledge practices would travel, particularly if they ignore the national civic epistemologies governing the interactions between science and society. Considerable institutional inertia exists to keep doing climate scenarios in the same way. Only the British radically changed their way it produced and communicated its climate scenarios between its last and most recent set, as Met Office Hadley Centre scientists pushed for greater innovation in its climate modelling. Whether the ‘Europeanisation’ of climate knowledge is possible or even undesirable remains open to debate (see Demeritt et al. 2013). Lastly, more research is needed to reconcile the contrasting experiences of scientists and users to better understand why good science is constructed differently and the implications this has. For instance, after consulting seemingly the same water users, why did UKCP09 and KMNI’14 scientists take radically different approaches to their climate scenarios? Different epistemic cultures alone cannot fully explain this. Indeed, user preferences over risk, politics and decision-making are powerful catalysts as well. Only by tracing the experiences of scientists and users together will we be able to fully understand what shapes climate information.

## Conclusion

Our research maps how different social and scientific values, and different institutional arrangements, shaped three sets of national climate scenarios. What knowledge is produced, how scientists and users interact and what the *user* expected to apply the climate scenarios are strongly influenced by the political culture of each country and the respective roles played by science, government and non-state organisations in each. Efforts to co-produce climate knowledge are restricted, possibly even counter-productive, if scientists are unwilling to listen to users in the first place. And, while new actors may join or user needs develop, producers and brokers of climate information need to be aware of, and responsive to, the political culture that incentivises such changes. While government-approved science may help improve the legitimacy and credibility of climate information, the same is not necessarily true for its saliency and usability. This insight has important implications for how societies will adapt to climate change and the extent to which their decisions will be effective.

## Footnotes

1 For clarity purposes, we use ‘climate scenarios’ to encompass ‘climate change scenarios’, ‘national climate assessments’ and ‘climate change projections’.

2 UKCP09 included numerous distinct products, such as marine scenarios, observed trends and a weather generator. We have analysed only the land scenarios and refer to this product when we use the term ‘UKCP09’.

3 Government approval played a key role in all three national climate scenarios we studied. Such approval represented national governments’ commitment to make as many people aware of climate change as possible and the need for them to take action. It also symbolised the commitment of scientists to make a difference to society by ensuring their work informed decision-making. Whereas UKCP09 and KNMI’14 emerged from pre-existing government-sponsored contracts, CH2011 was driven by scientists themselves and was only given a government approval prior to publication as it tied into wider political objectives including the Swiss Federal Councils’ strategy on climate adaptation.
